# Optimizing Neuromuscular Electrical Stimulation Pulse Width and Amplitude to Promote Central Activation in Individuals With Severe Spinal Cord Injury

**DOI:** 10.3389/fphys.2019.01310

**Published:** 2019-10-18

**Authors:** David J. Arpin, Beatrice Ugiliweneza, Gail Forrest, Susan J. Harkema, Enrico Rejc

**Affiliations:** ^1^Kentucky Spinal Cord Injury Research Center, University of Louisville, Louisville, KY, United States; ^2^Department of Neurological Surgery, University of Louisville, Louisville, KY, United States; ^3^Human Performance and Engineering Research, Kessler Foundation, West Orange, NJ, United States; ^4^Department of Physical Medicine and Rehabilitation, Rutgers New Jersey Medical School, Newark, NJ, United States; ^5^Frazier Rehab Institute, KentuckyOne Health, Louisville, KY, United States

**Keywords:** paralysis, recovery, neuromuscular electrical stimulation, neuromodulation, training, spinal cord injury

## Abstract

Neuromuscular electrical stimulation (NMES) is one of the most effective treatments for counteracting the deleterious skeletal muscle adaptations that occur after spinal cord injury (SCI). Additionally, previous findings suggest that NMES can activate motor units *via* both peripheral and central mechanisms; however, this NMES-promoted central activation is not well understood. In this study, we aimed at investigating the effects of NMES on central activation in 10 individuals with motor complete SCI, focusing on understanding how to optimize NMES pulse width and amplitude for promoting central activation in this population. To this end, we used NMES to generate isometric contractions of the knee extensors and ankle plantarflexors while electromyographic (EMG) activity was recorded from the vastus lateralis and gastrocnemius medialis, respectively. We used EMG activity that persisted after the termination of NMES delivery (post-NMES) as a neurophysiological marker to assess central activation and explored differences in post-NMES EMG activity promoted by 500 and 1,000 μs pulse width NMES. Additionally, we explored the relationships between post-NMES EMG amplitude, torque output, and stimulation amplitude. Our results show that the higher pulse width (1,000 μs) demonstrated a greater effect on central activation as quantified by more frequent occurrences of post-NMES EMG activity (*p* = 0.002) and a 3.551 μV higher EMG amplitude (*p* = 0.003) when controlling for the torque output generated by 500 and 1,000 μs pulse width NMES. Importantly, we also found that the interplay among central activation, stimulation amplitude, and muscle torque output differs across SCI individuals, conceivably because of the individual-specific characteristics of the cord lesion and following plasticity of the spinal circuitry. These results suggest that NMES can be optimized to promote central activation, which may lead to novel opportunities for motor function recovery after SCI.

## Introduction

Severe spinal cord injury (SCI) extensively alters the neuromuscular and skeletal systems below the level of injury, resulting in paralysis and deleterious skeletal muscle adaptations. For example, paralyzed muscles demonstrate an increase in the proportion of fast-fatigable motor units, as well as atrophy of all muscle fibers, resulting in increased fatigability and decreased force output, respectively (Shields, 1995, 2002). Neuromuscular electrical stimulation (NMES)-based training can be performed under isometric conditions (Shields and Dudley-Javoroski, 2006, 2007) as well as under more functional conditions (Mohr et al., 1997; Chilibeck et al., 1999; Crameri et al., 2002; Momeni et al., 2018) and is effective for promoting recovery of muscle mass and strength after SCI (Crameri et al., 2004; Shields and Dudley-Javoroski, 2006, 2007). Additionally, muscular stimulation has also been shown to be effective for increasing bone mineral density (Mohr et al., 1997; Lambach et al., 2018) and cardiovascular function (Gibbons et al., 2016; Shaffer et al., 2018). The selection of NMES parameters affects the characteristics of muscle activation. For example, the torque-frequency relationship exhibits a characteristic sigmoidal shape (Shields and Chang, 1997; Shields, 2002), which is due to the increase in stimulation frequency resulting in increased twitch summation, leading to tetanic contraction (Binder-Macleod and McDermond, 1992). Stimulation frequencies from 20 to 40 Hz are generally applied to elicit tetanic muscle contractions (Shields and Chang, 1997; Bigland-Ritchie et al., 2000). Likewise, NMES amplitude can be increased to elicit the progressive activation of motor units, resulting in greater force generation. It is interesting to note that NMES-induced motor unit recruitment order is still uncertain. One perspective suggests that NMES results in a random or nonselective motor unit recruitment, activating concurrently larger, fast motor units and smaller, slow motor units (Gregory and Bickel, 2005; Bickel et al., 2011). Alternatively, other studies support the view that NMES leads to a motor unit recruitment order where larger motor units, which generate higher force, are activated prior to the smaller motor units (Cabric et al., 1988; Trimble and Enoka, 1991; Heyters et al., 1994), representing an inverse activation order compared to voluntary contractions. Additionally, NMES is also characterized by a spatial recruitment of the muscle, primarily activating superficial muscle fibers (Vanderthommen et al., 2002). This superficial activation is due to the fact that NMES activates the axons which are in close proximity to the stimulating electrodes, and this recruitment diminishes proportionally with increasing distance from the electrodes (Vanderthommen et al., 1997, 2000). Finally, it has also been suggested that NMES may activate motor units *via* both peripheral and central mechanisms (Bergquist et al., 2011).

The fact that NMES may modulate the excitability of spinal circuitry to result in muscle contraction *via* central mechanisms (Bergquist et al., 2011) is of particular interest not only for the recovery of muscle mass and strength, but also for its potential to affect training-induced neural plasticity and consequent motor function recovery in individuals with SCI. In fact, other rehabilitative interventions such as spinal cord epidural stimulation and transcutaneous spinal stimulation are focused on modulating the excitability of lumbosacral spinal circuitry during activity-based training to promote long-lasting neural adaptations in this population (Gerasimenko et al., 2015; Rejc et al., 2017a,b; Wagner et al., 2018). While the effects of NMES pulse width on neuromuscular activation remain poorly understood, it is proposed that longer NMES pulse width may favor central (spinal) activation mechanisms (Collins, 2007). This is based on findings collected primarily from non-disabled individuals showing that wider pulse width NMES elicits greater muscle torque than narrower pulse widths, and that this increased muscle torque is abolished when an anesthetic nerve block is administered proximal to the stimulation site (Collins et al., 2001, 2002).

The aim of this study was to investigate the effects of NMES on central activation in individuals with motor complete SCI, with particular focus on understanding how to optimize NMES pulse width and amplitude for promoting central activation in this population. We hypothesized that wider pulse width NMES would promote greater central activation, and that inter-individual differences should be considered while selecting stimulation amplitudes for maximizing central activation.

## Materials and Methods

### Research Participants

Ten individuals with clinically motor complete SCI, graded A (*n* = 7) or B (*n* = 3) following the International Standards for Neurological Classification of Spinal Cord Injury (Burns et al., 2012), participated in this study. The average time since injury was 10.4 ± 9.8 years, and the level of injury ranged from cervical (C)2 to thoracic (T)6. The experimental protocol was approved by the Institutional Review Board at the University of Louisville and in accordance with the declaration of Helsinki. Additionally, all participants provided written informed consent prior to participating in this study.

### Experimental Protocol

Electrically stimulated isometric contractions were elicited for the knee extensors and ankle plantarflexors while subjects were seated in a dynamometer (Biodex Inc., Shirley, NY). These two muscle groups were selected as they are primary antigravity muscle groups. As the duration of the experimental protocol (approximately 1 h) allowed the testing of three muscle groups per research participant, the two muscle groups of the dominant leg, and one muscle group of the non-dominant leg were tested. The non-dominant knee extensors were tested in five participants, and the non-dominant ankle plantarflexors were tested in five participants. The chair of the dynamometer had the backrest set at an angle of 85°, with a knee angle of 110° for the knee extensor contractions. For the ankle plantarflexor contractions, the chair backrest was set at an angle of 55°, with an ankle angle of 95° (i.e., 5° of plantarflexion). Movement of the leg was minimized and the joint being tested was isolated by using two crossover shoulder straps, a belt across the lower abdomen, and a strap across the upper thigh. The torque output from the dynamometer was recorded by custom LabVIEW (National Instrument Inc., Austin, TX) software and sampled at 1 kHz.

NMES (Xcite Stimulator, Restorative Therapies, Inc., Baltimore, MD) was delivered to each of the muscle groups tested through two surface electrodes (Axelgaard Manufacturing Co., Ltd., Fallbrook, CA). Research participants were instructed to relax and not attempt to volitionally contract the muscles throughout the experiment. The NMES electrodes for knee extension were positioned over the quadriceps with the distal end of the proximal pad (5 × 9 cm) placed at 50% of the distance from the anterior superior iliac spine to the patella protrusion, and the distal end of the distal pad (5 × 9 cm) placed at 10% of the distance between the patella protrusion and the anterior superior iliac spine. The electrodes for plantarflexion were positioned over the triceps surae with the proximal pad (5 × 9 cm) placed at 30% of the distance from the tibial condyle to the lateral malleolus, and the distal pad (5 × 5 cm) placed at 30% of the distance from the lateral malleolus to the tibial condyle.

The relationship between stimulation amplitude and peak torque exerted (i.e., recruitment curve) (Arpin et al., 2018) was examined by delivering NMES starting with a stimulation amplitude of 5 mA, and increasing by 5 mA for every subsequent stimulation until either the participant requested to stop the stimulation due to discomfort, the maximum amplitude of the stimulator (140 mA) was reached, or the recruitment curve reached a plateau (at least 3 consecutive values with less than 5% difference). Each stimulation train consisted of a 0.5 s stimulation duration and 100 Hz stimulation frequency, which was delivered every 7 s. We selected 100 Hz as stimulation frequency because it was found more effective for eliciting central activation (Collins et al., 2002). These recruitment curves were generated twice for each muscle group, once with a 500 μs and once with a 1,000 μs NMES pulse width. The order of the two pulse widths was randomized across participants and muscle groups, and 3 min of rest was given between the two recruitment curve protocols. Additionally, to investigate the effects of pulse width on central activation, electromyography (EMG) (Delsys Inc., Natick, MA) was concurrently recorded from the vastus lateralis during knee extension stimulation and from the gastrocnemius medialis during plantarflexion stimulation. The EMG data were recorded by custom LabVIEW (National Instrument Inc., Austin, TX) software and sampled at 1 kHz.

### Data Analysis

All torque data were low-pass filtered at 10 Hz. For each NMES-induced muscle contraction, peak torque was defined as the maximum torque value.

Eight out of the 10 participants demonstrated EMG activity after the termination of NMES delivery (post-NMES), and were considered for further analysis, as this neurophysiological marker was assessed to examine afference-promoted central activation (Collins, 2007). To assess the effects of pulse width on post-NMES EMG amplitude and frequency characteristics, we compared the EMG data promoted by stimulation amplitudes that generated similar peak torque values while applying 500 and 1,000 μs pulse width. These similar peak torque values were identified manually. This allowed us to control for the larger muscle torque induced by the wider pulse width when the same stimulation amplitude was applied. All EMG data were notch filtered at 60 Hz, and band-pass filtered at 10–300 Hz. EMG amplitude was quantified by calculating the root mean square (RMS) normalized by baseline. Specifically, the baseline RMS value was calculated over a 500 ms time window selected from a quiet period between 1,650 and 150 ms prior to each 0.5 s stimulation train. This 1.5 s time period was visually inspected and the 500 ms window was selected for use as the baseline if no visible EMG response was present. This allowed the exact position of the baseline to be adjusted for individual contractions, and ensured that any visible EMG activity was avoided. The EMG baseline amplitude values selected in between muscle contractions were not greater that the EMG baseline amplitude +3 SD assessed prior to the beginning of stimulation. The baseline RMS value was then subtracted from the RMS calculated between 150 and 650 ms after each 0.5 s stimulation train. Additionally, the EMG median frequency was also calculated over this post-NMES 500 ms time window using Fast Fourier Transform. Finally, four representative patterns were observed when assessing the relationship among post-NMES EMG amplitude, torque output and stimulation amplitude, and were objectively characterized with the following criteria: 1) an initial increase in stimulation amplitude, torque output, and post-NMES EMG amplitude leading to the maximum EMG amplitude, followed by at least two consecutive decreases in EMG amplitude while further increasing stimulation amplitude, 2) concurrent increment of stimulation amplitude, torque output and post-NMES EMG amplitude, as indicated by statistically significant direct correlations among these variables; 3) Negligible torque output (< 3 Nm) with the presence of post-NMES EMG amplitude; and 4) No post-NMES EMG amplitude greater than the baseline mean + 3 standard deviations.

### Statistical Analysis

The distribution of EMG and torque variables was tested for normality using the Kolmogorov-Smirnov test, and the parametric or non-parametric tests reported below were applied accordingly. Differences in muscle torque output, post-NMES EMG amplitude and median frequency due to the application of 500 or 1,000 μs pulse width stimulation were statistically analyzed by generalized linear nested mixed model including random intercept for each experiment and random intercept and slope for each participant nested within experiment, which accounts for the variability resulting from the fact that multiple muscle groups were tested within the same individual. Pulse width was the only independent variable in the model. To obtain the estimated difference, we built a linear contrast on this variable. The results were presented in absolute difference with associated standard error. Additionally, Wilcoxon test was applied to determine if 1,000 μs pulse width stimulation promoted a significantly greater number of post-NMES EMG responses compared to 500 μs pulse width stimulation. Also, correlation between stimulation amplitude and EMG amplitude or peak torque normalized by their maximum values assessed within each subject and muscle group was tested by non-parametric Spearman rho rank order correlation. Finally, correlation analyses among post-NMES EMG amplitude, torque output and stimulation amplitude were performed in order to define one of the four representative individual patterns observed, which was characterized by the concurrent increment of these three variables. Statistical analysis was performed using SAS 9.4; all tests were two-sided and the alpha level was 0.05.

## Results

**Figure 1:**
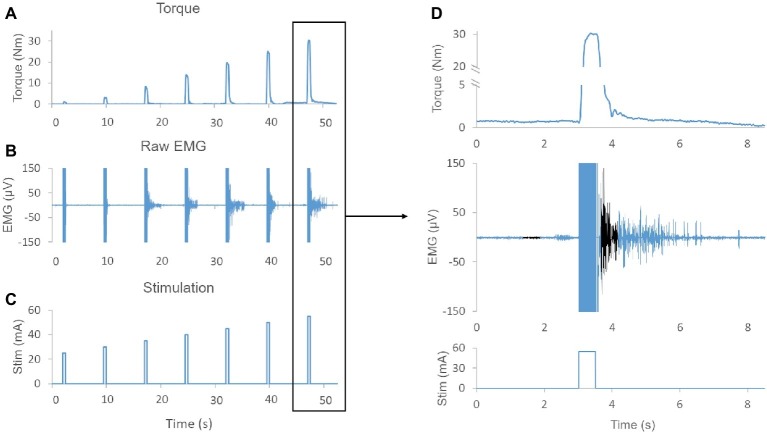
**(A)** Exemplary muscle torque output elicited by increasing stimulation amplitudes (i.e., recruitment curve). **(B)** Exemplary raw EMG showing sustained EMG activity after termination of NMES for several evoked contractions. **(C)** NMES protocol demonstrating the amplitude and duration of the stimulation. **(D)** Magnification of torque output, raw EMG, and stimulation pulse. Note, EMG activity is sustained for more than 2 s after termination of the stimulation. The baseline 500 ms time window and the 500 ms time window used to quantify post-NMES EMG amplitude are shown in black. Also, the negligible (0.7 Nm) offset in the baseline torque signal was due to a lateral weight shift of the research participant.

During the generation of recruitment curves for knee extensors and plantar flexors, NMES promoted the generation of EMG activity that persisted after the end of stimulation in 8 out of the 10 individuals tested (Figure 1). The total number of elicited contractions that demonstrated a post-NMES EMG response was significantly greater for the 1,000 μs pulse width than the 500 μs pulse width (*p* = 0.002). In particular, 500 μs stimulation elicited post-NMES EMG activity in 235 out of 705 muscle contractions initially examined (33.3%), while 1,000 μs pulse width promoted post-NMES EMG activity in 288 out of 704 muscle contractions initially considered for analysis (40.9%).

**Figure 2:**
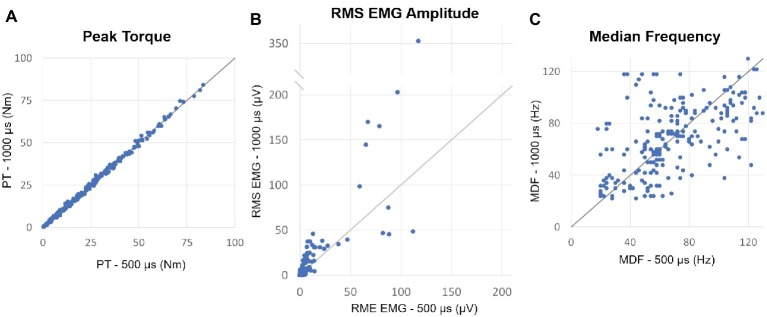
**(A)** Plot of the peak torques (PT) from contractions induced by 500 vs. 1,000 μs pulse widths which generated similar muscle torque output and elicited post-NMES EMG activity. The identity line is also shown and indicates that peak torque did not differ between matched contractions induced by the two pulse widths. **(B)** Plot of the post-NMES EMG RMS value for 500 vs. 1,000 μs pulse widths, as well as the identity line. The majority of the RMS values are above the identity line, indicating that 1,000 μs pulse width resulted in greater post-NMES EMG activity. **(C)** Plot of the median frequency (MDF) of post-NMES EMG activity for 500 vs. 1,000 μs pulse width, with the identity line. The data appear evenly distributed about the identity line.

For each individual, we selected for further analysis muscle contractions induced by both 500 and 1,000 μs stimulation that generated similar muscle torque output and elicited post-NMES EMG activity. The muscle contractions generated from NMES at 500 and 1,000 μs pulse width were appropriately matched, as torque output was very similar for all paired contractions (mean difference = 0.072 ± 0.226 Nm) and no significant differences (*p* = 0.320) were observed between muscle torque induced by 500 and 1,000 μs pulse width (Figure 2A). On the other hand, post-NMES EMG amplitude promoted by 1,000 μs pulse width was 3.551 ± 1.19 μV (+63%) larger than that elicited by 500 μs pulse width (*p* = 0.003), indicating that 1,000 μs pulse width resulted in greater central activation when muscle torque output was matched (Figure 2B). Conversely, no significant difference was found in the median frequency of post-NMES EMG activity promoted by 500 and 1,000 μs pulse width (Figure 2C; mean difference = 0.089 ± 1.453 Hz; *p* = 0.951).

**Figure 3:**
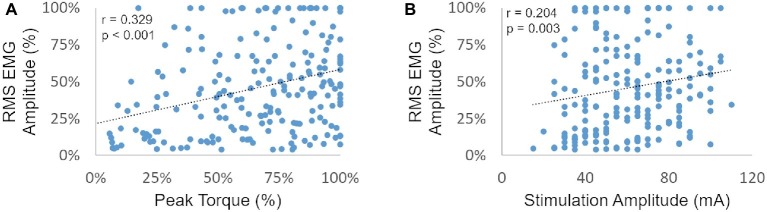
RMS EMG amplitude (as a percent of the maximum generated within each subject and muscle group) is plotted against the related peak torque [expressed as a percent of the maximum peak torque generated within each subject and muscle group **(A)** and against stimulation amplitude **(B)**].

We then plotted the post-NMES EMG amplitude promoted by 1,000 μs pulse width against muscle torque output and NMES amplitude to assess whether these variables were significantly correlated (Figures 3A,B, respectively). In particular, EMG amplitude and peak torque were normalized by their maximum value assessed within each subject and muscle group.

**Figure 4:**
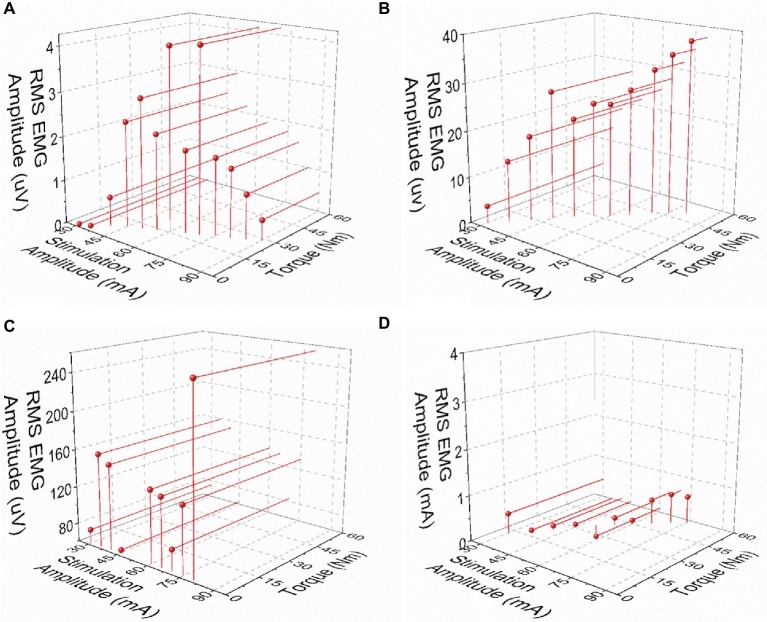
Exemplary patterns representing the relationships between post-NMES EMG amplitude, torque output, and stimulation amplitude. **(A)** Increases in stimulation amplitude and torque output, with maximal RMS EMG amplitude value at about mid-stimulation amplitude. **(B)** Concurrent increments in stimulation amplitude, torque output, and EMG amplitude. **(C)** Increase in stimulation amplitude with negligible torque output and large EMG amplitude values. **(D)** Negligible RMS EMG amplitude values with increases in stimulation amplitude and torque output.

Post-NMES EMG amplitude was found to be directly correlated with both peak torque and NMES amplitude (*p* < 0.01). On the other hand, the high residual variance in these correlations indicates that factors other than muscle torque output and stimulation amplitude play an important role in determining post-NMES EMG amplitude. In order to explore this topic further, we examined the individual relationships among post-NMES EMG amplitude, torque output, and stimulation amplitude, identifying the four representative patterns reported in Figure 4. The most common pattern, observed in 53% of the muscles tested, is exemplified in Figure 4A. This was characterized by an expected direct relationship between stimulation amplitude and peak muscle torque, while post-NMES EMG amplitude initially increased along with stimulation amplitude (i.e., 30–70 mA) and then progressively decreased for subsequent increments in stimulation amplitude (i.e., 75–90 mA). The second pattern, which was observed in 18% of the muscles tested, consisted in the concurrent increment of stimulation amplitude, torque output and EMG amplitude (correlation *p* values between <0.0001 and 0.0321; and *r* values between 0.998 and 0.537; Figure 4B). The third pattern was characterized by the presence of relevant post-stimulation EMG activity even if negligible muscle torque was generated (Figure 4C; 11% of the muscles tested). Finally, 18% of the muscle groups tested did not generate any EMG activity in response to NMES (Figure 4D).

## Discussion

In this study, we examined the effects of NMES pulse width on central activation in individuals with motor complete SCI. We demonstrated that wide pulse (500 and 1,000 μs) NMES elicits EMG activity that persists after the end of stimulation (Figure 1) in SCI individuals. This post-NMES EMG activity indicates afference-promoted central activation leading to motor unit recruitment (Collins, 2007). Interestingly, the higher pulse width (1,000 μs) demonstrated a greater effect on central activation as quantified by the more frequent occurrence of post-NMES EMG activity and the larger EMG amplitude promoted by NMES-induced muscle contractions that generated similar torque output (Figures 2A,B). This finding directly shows that higher pulse width NMES is more effective for promoting greater central activation in motor complete SCI individuals, and supports previous findings showing that high pulse width elicits extra torque and post-stimulation EMG activity (Collins et al., 2001, 2002; Nickolls et al., 2004; Collins, 2007). The mechanisms behind NMES-promoted central activation are not well understood; however, they are presumed to be related to the activation of sensory axons (Collins et al., 2001, 2002). This theory is supported by evidence that shorter pulse width stimulation primarily activates motor axons (Grill and Mortimer, 1996), while longer pulse width stimulation activates more sensory axons due to the lower rheobase and longer strength-duration constant of sensory axons compared to motor axons (Veale et al., 1973; Mogyoros et al., 1996). Once the sensory volley enters the spinal cord, it may activate motoneuron plateau potentials through voltage-gated Ca^++^ channels that may produce persistent inward currents, causing continuous depolarization (Hultborn, 2002; Heckmann et al., 2005; Heckman et al., 2008), and thus self-sustained motoneuron discharge (Klakowicz et al., 2006).

In an attempt to gain further insight on the mechanisms behind NMES-elicited central activation, we also assessed the median frequency of post-NMES EMG activity promoted by 500 and 1,000 μs NMES. Interestingly, when muscle contractions that generated similar torque output were compared, NMES pulse width did not affect EMG median frequency (Figures 2A,C). Taken together, the findings herein reported suggest that the greater central activation promoted by wider pulse width results in the recruitment of more motor units without altering the average conduction velocity of the muscle fibers assessed by surface EMG (Farina et al., 2004).

We also found weak but significant positive correlations between post-NMES EMG amplitude and peak torque elicited by NMES as well as stimulation amplitude (Figure 3). Increased afferent input to the spinal circuitry related to the activation and increased activity of different receptors (i.e., muscle spindles; Golgi tendon organ) (Blum et al., 2017) along with larger muscle torque output may contribute to a higher central activation (Gerasimenko et al., 2015). Likewise, increased stimulation amplitude may lead to greater activation of afferent fibers (Collins et al., 2001, 2002), thus resulting in increased central excitability. Interestingly, the high residual variance in the correlations reported in Figure 3 indicates that factors other than muscle torque output and stimulation amplitude play an important role in determining post-stimulation EMG amplitude. In particular, our analysis suggests that the interplay among central activation, stimulation amplitude and muscle torque output can be different across SCI individuals (Figure 4), conceivably because of the individual-specific characteristics of the cord lesion and following plasticity of the spinal circuitry (Hiersemenzel et al., 2000; Beauparlant et al., 2013). Interestingly, the most common pattern resulted in the direct relationship between stimulation amplitude and torque output, while post-NMES EMG amplitude initially increased along with stimulation amplitude and then progressively decreased for subsequent increments in stimulation amplitude (Figure 4A). This inhibitory effect on central activation may be caused by an antidromic block. In fact, higher simulation amplitudes increase the antidromic transmission in motor axons, which block the orthodromic action potentials that arise within the spinal cord, reducing the central recruitment of motor units (Gottlieb and Agarwal, 1976; Pierrot-Deseilligny and Mazevet, 2000). Additionally, as central activation has been hypothesized to be related to the production of persistent inward currents, the individual-specific patterns we observed may also be due to differences in the levels of monoamines present in the spinal cord, which have previously been linked to variability in the prevalence of persistent inward currents in animals (Harvey et al., 2006). Another recurrent pattern was characterized by the concurrent increment of stimulation amplitude, torque output, and EMG amplitude (Figure 4B). It is possible that the decrease in post-NMES EMG related to the antidromic block would have occurred at stimulation amplitudes higher than those allowed by the experimental conditions for these participants and muscle groups. Finally, the third common pattern consisted in the detection of post-NMES EMG activity while negligible muscle torque was generated (Figure 4C), suggesting that higher stimulation amplitudes can promote central activation even without mechano-related sensory input contributing to increased spinal excitability. The fact that we did not detect any post-NMES EMG in two of the tested individuals (i.e. Figure 4D) is consistent with previous observations (Nickolls et al., 2004), which also showed that it may be easier to elicit signs of central activation in non-disabled as compared to SCI individuals. In particular, it was suggested that the inability to promote central activation in some individuals with SCI might be related to the presence of peripheral nerve dysfunction or limitations in the stimulation amplitude, while no correlation between clinical characteristics (level or completeness of injury) and the ability to evoke signs of central activation was found (Nickolls et al., 2004). Additionally, as mentioned above, inter-individual differences in the neurobiological state of the spinal cord may also contribute to the variability in promoting central activation in individuals with SCI.

Limitations of this study include the relatively small sample size, which may not characterize this population as a whole, and the absence of individuals with severe although clinically incomplete SCI. Additionally, only two different pulse widths (500 and 1,000 μs) were assessed to explore the effect on central activation. Future studies, among others, should investigate whether pulse widths greater than 1,000 μs may promote even greater central activation, and whether the pulse width promoting greater central activation is individual-specific. However, the fact that NMES pulse width and amplitude can be optimized to promote central activation may lead to novel opportunities for motor function recovery after SCI. Severe SCI leads to the inability to walk and stand primarily because of the resulting loss of tonic supraspinal drive to the spinal circuits controlling posture and locomotion, which compromises their state of excitability (Harkema, 2008; Cote et al., 2017). Interestingly, recent studies have shown that the combination of activity-based training and spinal cord stimulation aimed at modulating the spinal cord excitability can promote long-lasting neural adaptations and motor function improvements that can persist after spinal stimulation (Gerasimenko et al., 2015; Rejc et al., 2017a,b; Wagner et al., 2018). Here, we propose that future studies should apply NMES parameters tailored to maximize spinal circuitry activation (using higher pulse width and individual-specific amplitude modulation) and define the optimal timing for administering stimulation while performing activity-based training, so that the related sensory feedback can be effectively processed by the spinal circuitry (and not overpowered by electrical stimulation) to result in an enhanced training-induced neural plasticity. Hence, improved motor recovery could be achieved while concurrently taking advantage of the important NMES-promoted muscle and bone adaptations.

## Data Availability Statement

The datasets generated for this study are available on request to the corresponding author.

## Ethics Statement

The studies involving human participants were reviewed and approved by the Institutional Review Board at the University of Louisville. The patients/participants provided their written informed consent to participate in this study.

## Author Contributions

ER and DA contributed to the experimental design, data analysis, and data collection. DA wrote the first draft of the manuscript. BU contributed to the statistical analysis and interpretation of results. ER, GF, and SH contributed to interpretation of the results. All authors revised the manuscript and approved its final version.

### Conflict of Interest

The authors declare that the research was conducted in the absence of any commercial or financial relationships that could be construed as a potential conflict of interest.
